# Quantitative Evaluation of Scintillation Camera Imaging Characteristics of Isotopes Used in Liver Radioembolization

**DOI:** 10.1371/journal.pone.0026174

**Published:** 2011-11-03

**Authors:** Mattijs Elschot, Johannes Franciscus Wilhelmus Nijsen, Alida Johanna Dam, Hugo Wilhelmus Antonius Maria de Jong

**Affiliations:** 1 Department of Radiology and Nuclear Medicine, University Medical Center Utrecht, Utrecht, The Netherlands; 2 Department of Medical Technology, Gelre Hospital, Apeldoorn, Gelderland, The Netherlands; Genentech, United States of America

## Abstract

**Background:**

Scintillation camera imaging is used for treatment planning and post-treatment dosimetry in liver radioembolization (RE). In yttrium-90 (90Y) RE, scintigraphic images of technetium-99m (99mTc) are used for treatment planning, while 90Y Bremsstrahlung images are used for post-treatment dosimetry. In holmium-166 (166Ho) RE, scintigraphic images of 166Ho can be used for both treatment planning and post-treatment dosimetry. The aim of this study is to quantitatively evaluate and compare the imaging characteristics of these three isotopes, in order that imaging protocols can be optimized and RE studies with varying isotopes can be compared.

**Methodology/Principal Findings:**

Phantom experiments were performed in line with NEMA guidelines to assess the spatial resolution, sensitivity, count rate linearity, and contrast recovery of 99mTc, 90Y and 166Ho. In addition, Monte Carlo simulations were performed to obtain detailed information about the history of detected photons. The results showed that the use of a broad energy window and the high-energy collimator gave optimal combination of sensitivity, spatial resolution, and primary photon fraction for 90Y Bremsstrahlung imaging, although differences with the medium-energy collimator were small. For 166Ho, the high-energy collimator also slightly outperformed the medium-energy collimator. In comparison with 99mTc, the image quality of both 90Y and 166Ho is degraded by a lower spatial resolution, a lower sensitivity, and larger scatter and collimator penetration fractions.

**Conclusions/Significance:**

The quantitative evaluation of the scintillation camera characteristics presented in this study helps to optimize acquisition parameters and supports future analysis of clinical comparisons between RE studies.

## Introduction

Intra-arterial liver radioembolization (RE) using microspheres loaded with a high-energy beta emitting isotope, is an emerging technique for radiation treatment of patients with unresectable liver tumors [1], [2]. RE leads to high tumor absorbed radiation doses, while the surrounding healthy liver tissue is spared [3]. RE is generally a two-step procedure, consisting of i) the administration of a small amount of activity, the scout dose, and ii) subsequent administration of the activity that is expected to have a therapeutic effect, the therapy dose. The spatial distribution of the scout dose is expected to represent the distribution of the therapy dose, and can consequently be used to predict safety of the procedure.

The spatial distribution of the scout and therapy dose is commonly assessed using scintillation camera imaging. Following administration of the scout dose, the patient undergoes single photon emission computed tomography (SPECT) to detect extrahepatic deposition of activity, which is a contra-indication for therapy, and planar scintillation camera imaging to determine the percentage of activity that shunted to the lungs. A high lung-shunting (LS) percentage provokes reduction of the therapy dose (10%<LS<20% (SIR-Spheres package insert; Sirtex Medical, Sydney, Australia)) or even cancellation of therapy (LS>20%). After treatment, a SPECT scan of the therapy dose is usually acquired to confirm absence of extrahepatic activity. This scan can also be used to retrospectively assess a dose-response relation.

The beta-emitter yttrium-90 (^90^Y, Table 1) is most frequently used for RE. Its distribution can be assessed by means of SPECT imaging of the Bremsstrahlung photons [4]–[7]. Unfortunately, the continuous Bremsstrahlung energy spectrum comprises photon energies ranging up to 2 MeV, and is therefore associated with poor image quality [7]. For this reason, Technetium-99m-labeled macroaggregated albumin particles (^99m^Tc-MAA) are generally used for the scout dose, as a substitute for the ^90^Y microspheres, since the image quality is much better [8], [9]. The ^99m^Tc-MAA particles, however, differ in shape, size, density and quantity from the ^90^Y microspheres, potentially affecting the spatial distribution of the scout dose [10], [11]. Furthermore, the instability of ^99m^Tc-MAA particles frequently leads to uptake of dissociated ^99m^Tc-pertechnetate in the thyroid and gastrointestinal tract [12], and to shunting of smaller and disintegrated particles to the lungs [13], which altogether may cause false adjustment of the ^90^Y therapy dose. As a potential alternative to ^90^Y microspheres, holmium-166 (^166^Ho, Table 1) loaded microspheres are currently investigated in a clinical study [14]–[18]. The higher specific activity and shorter half life may have a beneficial impact on tumor kill. Additionally, the imaging characteristics of ^166^Ho are believed to be more suitable for quantitative imaging than ^90^Y, because it is a combined beta-gamma emitter [19], [20]. ^166^Ho microspheres may conceivably be used for both the scout dose and the therapy dose, which potentially improves the predictive value of the scout dose distribution and post-therapy liver dosimetry.

**Table 1:** Radioisotope characteristics and measurement settings.

Isotope	Half-life [h]	E_βmax_ [MeV]^a^	E_γ_ [keV]^b^	E_win_ [keV]^c^	Collimator
**^99m^Tc**	6.0	N/A	140.5 (89%)	130–151	VXGP
**^90^Y**	64.1	2.28 (99.9%)	Bremsstrahlung	120–250	MEGP
				50–250	HEGP
**^166^Ho**	26.8	1.77 (48.7%)	80.6 (6.7%)	74.6–86.6	MEGP
		1.85 (50.0%)	1379.4 (0.9%)		HEGP
			1581.0 (0.2%)		

a E_βmax_ represents the maximum energy and abundance of the beta particles.

b E_γ_ represents the gamma photon energy and abundance.

c E_win_ represents the lower and upper limits of the energy window.

Scintillation camera images of ^99m^Tc, ^90^Y, and ^166^Ho are all used for clinical assessment of the activity distribution in liver RE, but they have differing imaging characteristics. In contrast to ^99m^Tc, information on the scintillation camera imaging characteristics of ^90^Y and ^166^Ho is sparse, and a systematical comparison between these three isotopes does not exist. Therefore, the aim of this study is to provide a systematical evaluation and comparison of the scintillation camera imaging characteristics of ^90^Y, ^166^Ho and ^99m^Tc, using a series of phantom experiments and Monte Carlo simulations. This is important for (future) clinical comparisons between microsphere RE studies with varying isotopes and imaging protocols. In addition, the outcome of this study can be used to optimize the acquisition settings for ^90^Y and ^166^Ho imaging, and to guide future developments in SPECT image reconstruction.

## Materials and Methods

The spatial resolution, sensitivity, count rate linearity, and contrast recovery of ^99m^Tc, ^90^Y, and ^166^Ho were evaluated by means of phantom experiments. If possible, the well-defined National Electrical Manufacturers Association (NEMA) 2007 guidelines were adopted, facilitating (future) comparison with other isotopes and acquisition protocols [21], [22]. In addition, Monte Carlo simulations were performed to yield data that cannot be retrieved from physical experiments, including the fractions of primary photons, scattered photons, and collimator-penetrated photons of the line source measurements.

### Phantom experiments

Data was acquired with a Philips FORTE™ dual-headed camera with 3/8 inch thick thallium-doped sodium-iodide (NaI(Tl)) crystals. ^99m^Tc measurements were performed with the low-energy general purpose (VXGP) collimator. Medium-energy (MEGP) and high-energy general purpose (HEGP) collimators were mounted for the ^90^Y and ^166^Ho measurements, to reduce collimator penetration of high-energy photons. The energy window settings for ^90^Y Bremsstrahlung imaging vary between institutions, ranging from small windows with a relatively low central energy [23], [24], to intermediate [5], [6] and broad energy windows [4], [7], [25]–[27]. Although it is generally believed that a wide energy window is required to maintain sufficient sensitivity, no consensus exists on the optimal central energy and window width. Therefore, both a broad 50–250 keV window, which optimizes sensitivity, and a smaller 120–250 keV window, which is expected to have less scatter contamination, were evaluated in this study. The used energy window settings and collimator characteristics are listed in Table 1 and 2, respectively.

**Table 2:** Collimator characteristics.

Collimator	Hole size (mm)	Septal thickness (mm)	Length (mm)
**VXGP**	1.78	0.152	42
**MEGP**	2.95	1.143	48
**HEGP**	3.81	1.727	60

All collimators have hexagonal hole shapes.

#### Spatial resolution

**Figure 1:**
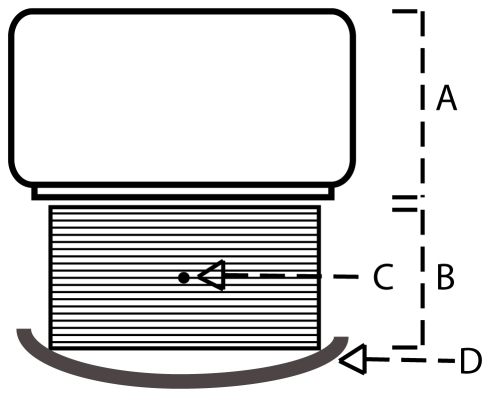
Schematic overview of the spatial resolution measurement of the line-source centered in 20 cm PMMA. Shown are the camera, including the collimator (A), a stack of 20 PMMA slabs of size 40×40×1 cm (B), the location of the line-source (C), and the patient bed (D). The line-source to collimator-face distance is 11 cm.

Spatial resolution defines whether small accumulations of activity, e.g. in or around tumors, can be detected and quantified. Projection images (matrix = 256×256, pixelsize = 2.332×2.332 mm^2^) of a 28 cm long line source with an inner diameter of 2 mm were acquired. To create realistic conditions with regard to electron absorption, and (Bremsstrahlung) photon emission and scatter, the line source was centered in 2, 10, and 20 cm of poly(methyl methacrylate) (PMMA), leading to 1, 5, and 10 cm of scatter material below and on top of the source. Measurements were performed with a source to collimator distance of 2, 6 and 11 cm for 1 cm of scatter material and 11 cm for the measurements with 5 and 10 cm of scatter material (Figure 1). The central lines perpendicular to the source were summed over a distance of 10 cm to obtain the line spread function (LSF). The full width at half maximum (FWHM) and the full width at tenth maximum (FWTM) of the LSF were determined according to NEMA guidelines.

#### Sensitivity

The imaging sensitivity of an isotope is directly related to the image noise, which can be important for detection of small amounts of extrahepatic activity. As described by NEMA, a Petri dish was filled with a thin layer of activity, which was beforehand measured with a calibrated dose calibrator. The dish was placed in the center of the field-of-view of the camera, at a distance of 10 cm and 40 cm from the collimator. To ensure stopping all electrons and proper creation of the Bremsstrahlung photons, 1 cm thick PMMA slabs were placed below and on top of the dish for the ^90^Y and ^166^Ho sensitivity measurements. In each measurement, 4 million counts were acquired. The sensitivity (S) [cps Mbq^−1^] was calculated as the total number of counts in the field-of-view, divided by acquisition time times activity.

To enable calculation of the system count-rate linearity, described in the next section, the sensitivity measurements were simultaneously acquired in two energy windows: the regular photopeak energy window, and an energy window covering the complete energy range of the camera (‘full-energy-window’).

#### Count rate linearity

In RE, therapy doses up to several GBqs are typically administered to the patient. These high activities can lead to detector dead-time and, consequently, to underestimation of the dose. This effect is described by the system count rate curve, that depends on i) the system sensitivity, which is determined by the number of photons that pass the collimator and fall within the energy window, and ii) the dead-time, which depends on the total number of photons that hit the crystal. The first can be measured as described in the ‘sensitivity’ section. The latter is theoretically described by Sorensen's count rate model for paralyzable scintillation cameras [28] and can be provided by the intrinsic count rate measurements described here.

Cylindrical vials with an inner diameter of 2.4 cm were filled with the isotopes solved in 11 ml water. Activities ranged from 0.5–24.5 MBq for ^99m^Tc, 16.9–350.8 MBq for ^90^Y and 5.0–121.1 MBq for ^166^Ho, to include count rates from the linear range to count rates that cause dead-time effects. The vials were placed in the center of the field-of-view of the camera, at 66.5 cm from the detector, resulting in a uniform photon flux on the detector. The acquisition time was 10 minutes and the energy window was set to the full-energy-window, yielding an intrinsic count rate [cps] comprised of all photons that hit the detector.

From the intrinsic count rate curve (measured in the full-energy-window), the system sensitivity in the full-energy-window, and the system sensitivity in the photopeak window, the system count rate in the photopeak window can be calculated for each isotope and collimator and plotted as a function of activity. The reported maximum activity with linear count rate response (A_linmax_) was defined as the highest activity with less than 2% loss of count rate. The corresponding maximum achievable linear count rate (R_linmax_) was also reported.

#### Contrast recovery

**Figure 2:**
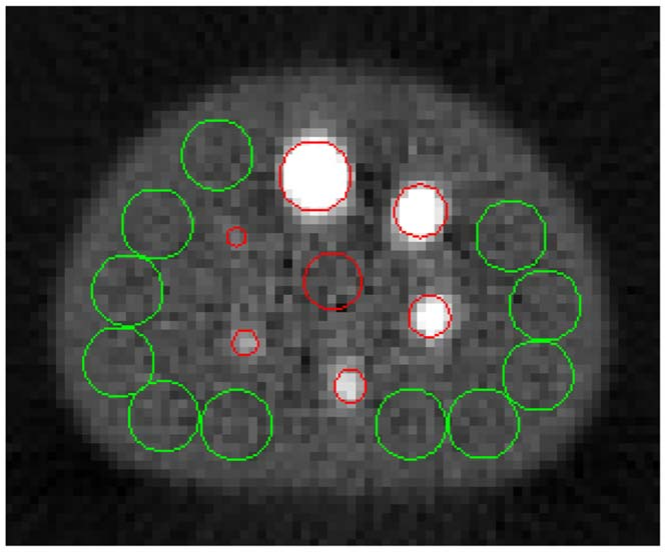
Overview of the hot sphere and background ROI. The slice through the center of the spheres of the contrast recovery phantom filled with ^99m^Tc is shown. Overlaid are the locations of the lung insert (central red ROI), the hot sphere ROI (peripheral red ROI), and 11 of the 55 background ROI of the largest sphere (green ROI).

Contrast recovery is important if quantification of local accumulation of activity is required, as is the case with image based tumor dosimetry in RE. Contrast recovery was determined using a fillable torso-shaped NEMA image quality phantom (volume = 9700 ml) containing six fillable coplanar spheres (inner diameter = 10, 13, 17, 22, 28, and 37 mm). Lung tissue was simulated by a central cylindrical lung insert. The phantom background activity concentrations and SPECT scan times were matched to obtain the total number of counts corresponding to a 30 minutes SPECT scan with clinically realistic liver activity concentrations: 137 kBq ml^−1^, 573 kBq ml^−1^, and 166 kBq ml^−1^, for ^99m^Tc, ^90^Y, and ^166^Ho, respectively, and a sphere-to-background activity concentration ratio (R) of 9∶1. These activity concentrations were based on a scout dose for ^99m^Tc and^166^Ho, and a therapy dose for ^90^Y. SPECT data was acquired in 120 projections over a 360° orbit. Volumes were reconstructed using filtered back-projection (FBP), on a 128×128×128 grid with an isotropic voxel size of 4.664 mm. No additional filtering other than the ramp filter was applied. Post-reconstruction, images were corrected for attenuation using the Chang algorithm with a non-uniform effective broad beam linear attenuation coefficient. Contrast recovery coefficients (QH) were calculated in accordance with NEMA guidelines. An example of the filled phantom with hot sphere and background regions of interest (ROI) is given in Figure 2.

### Monte Carlo simulations

Monte Carlo simulations were performed with the well validated general purpose Monte Carlo radiation transport code mcnpx 2.5.0 [29]. The camera head was modeled realistically including collimator housing, collimator, aluminum crystal housing, NaI(Tl) crystal, 5 cm of crown glass mimicking the backscatter compartments, and lead shielding. Both gamma photon (for ^99m^Tc and ^166^Ho) and Bremsstrahlung photon (for ^90^Y and ^166^Ho) contributions were simulated. The mono-energetic gamma photons were simulated weighted by their relative abundance as given in Table 1. Only 2.0% of the ^90^Y and 1.2% of the ^166^Ho beta emissions lead to Bremsstrahlung photons with an energy higher than 50 keV. Therefore, the Bremsstrahlung photons were sampled from a predefined energy spectrum and source distribution, similar to the approach published by Rault et al [30], which improved the efficiency of the simulations substantially.

#### Primary, scatter, and penetration fraction

Monte Carlo simulations were performed to estimate the fractions of primary (PF), scattered (SF) and collimator-penetrated (CPF) photons. Since high SF and CPF have deteriorating effects on the image quality, their characterizations give insight into the effectiveness of the chosen collimator and energy window. The geometry of the simulations was equal to the geometry of measurements of the line source centered in 20 cm PMMA, shown in Figure 1. A detected photon was defined as a photon that deposited at least part of its energy in the crystal, sub-divided in three groups:

- Primary photons: detected photons that did not interact with material or penetrate through the collimator.
- Scattered photons: detected photons that scattered in the PMMA, photons that scattered in the camera-housing or detector, and photons that were created in the collimator (lead x-rays). None of these photons penetrated the collimator.
- Penetrated photons: all detected photons (unscattered or scattered) that penetrated through the collimator.

The number of photons released was 300 million for both gamma photon and Bremsstrahlung photon simulations. Equal to the spatial resolution measurements, the central lines perpendicular to the source were summed over a distance of 10 cm to obtain the LSF. The total ^166^Ho LSF was calculated as the abundance weighted summation of gamma and Bremsstrahlung photon contributions. The experimental and simulated LSF measurements were compared visually.

## Results

### Phantom experiments

#### Spatial resolution

Table 3 lists the FWHM and FWTM in mm of the measured LSF for the three isotopes. In both energy windows, the ^90^Y LSF showed smaller FWHM and FWTM for the HEGP than for the MEGP collimator. The FWHM were slightly smaller for the 120–250 keV window than for the 50–250 keV window, but the FWTM were substantially larger. For ^166^Ho, the HEGP collimator yielded a higher resolution than the MEGP collimator, as can be appreciated by the slightly smaller FWHM, and substantially smaller FWTM, for all measurements.

**Table 3:** Spatial resolution given as the FWHM (FWTM) in mm.

	^99m^Tc	^90^Y 120–250 keV		^90^Y 50–250 keV		^166^Ho	
	VXGP	MEGP	HEGP	MEGP	HEGP	MEGP	HEGP
**S01D02**	5.4 (10.4)	11.5 (173.7)	7.0 (113.1)	10.9 (123.2)	7.5 (49.2)	11.1 (61.7)	6.5 (17.2)
**S01D06**	6.6 (12.3)	12.9 (222.2)	11.0 (137.2)	11.4 (149.7)	11.1 (60.2)	11.7 (42.2)	10.5 (21.5)
**S01D11**	8.2 (15.0)	17.1 (286.1)	15.3 (160.0)	15.8 (172.9)	15.4 (57.6)	14.7 (35.8)	14.1 (25.4)
**S05D11**	8.4 (16.6)	19.7 (300.6)	16.4 (218.9)	19.4 (235.7)	17.0 (159.3)	15.8 (97.0)	14.6 (43.5)
**S10D11**	8.5 (17.4)	28.1 (341.2)	18.1 (269.5)	26.3 (294.9)	20.1 (241.3)	15.6 (168.3)	14.9 (113.7)

S01D02 corresponds to the measurement with 1 cm of scatter material and line-source to collimator distance of 2 cm, S01D06 to the measurement with 1 cm of scatter material and line-source to collimator distance of 6 cm, etc.

#### Sensitivity

The planar sensitivities, measured at a distance of 10 and 40 cm from the collimator face, are listed in Table 4. As was expected from the photon abundances, the ^99m^Tc sensitivity was higher than the ^166^Ho sensitivity. The ^90^Y sensitivity was lower than ^166^Ho sensitivity for both energy windows. No difference was observed between the ^99m^Tc sensitivities at 10 cm and 40 cm, indicating proper collimation of the photons. To the contrary, the sensitivity for both ^90^Y and ^166^Ho decreased at a larger distance from the collimator, due to collimator penetration effects. This effect was larger for the MEGP collimator than for the HEGP collimator.

**Table 4:** Sensitivity in counts per second per unit activity.

	^99m^Tc	^90^Y 120–250 keV		^90^Y 50–250 keV		^166^Ho	
	VXGP	MEGP	HEGP	MEGP	HEGP	MEGP	HEGP
**S - 10 cm [cps MBq^−1^]**	63.8	6.0	3.1	10.5	6.0	12.6	10.6
**S - 40 cm [cps MBq^−1^]**	63.1	3.0	1.8	6.0	4.9	9.9	8.2

#### Count rate linearity

**Figure 3:**
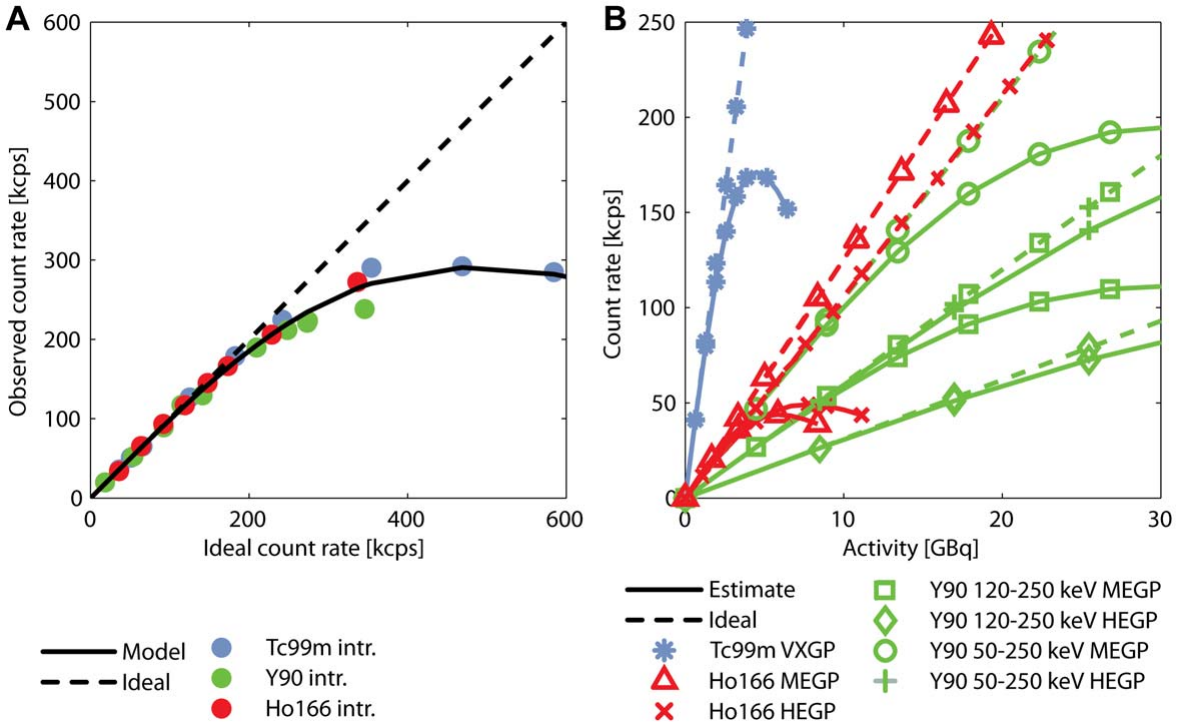
Intrinsic and system count rate linearity curves. (A) The observed count rate is plotted as a function of ideal count rate. Sorensen's count rate model for paralyzable cameras (solid line) is fitted to the intrinsic count rate measurements (data points). The ideal camera count rate response, without dead-time effects, is plotted by the dashed line. (B) Shown are the system count rate curves (solid lines), composed of the intrinsic count rate linearity curve and the system sensitivity measurements. The slope of the ideal system camera count rate response (dashed lines) corresponds to the system sensitivity.

Figure 3A shows the observed intrinsic count rate as a function of the ideal count rate for all isotopes. As expected, this intrinsic count rate curve did not depend on the isotope imaged and was fitted well by Sorensen's count rate model of paralyzable scintillation cameras [28]. Figure 3B shows the system count rate curves for all isotopes and measurement settings, estimated from the intrinsic count rate curve and the system sensitivities. A_linmax_ and R_linmax_ are listed in Table 5. The A_linmax_ of approximately 1.5 GBq for ^166^Ho might be a limitation for scintillation camera imaging directly after treatment. The quantitative accuracy of imaging the ^99m^Tc-MAA scout dose (∼150 MBq), ^166^Ho scout dose (∼250 MBq), and ^90^Y therapy dose (∼1–3 GBq) in the clinical range will not be affected by dead-time effects.

**Table 5:** Estimated maximum activity in the linear detection range and corresponding estimated maximum linear count rate.

	^99m^Tc	^90^Y 120–250 keV		^90^Y 50–250 keV		^166^Ho	
	VXGP	MEGP	HEGP	MEGP	HEGP	MEGP	HEGP
**A_linmax_ [MBq]**	1095	7595	14430	7595	14430	1420	1891
**R_linmax_ [kcps]**	68	45	44	78	85	18	20

#### Contrast recovery

**Figure 4:**
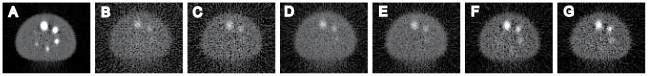
FBP reconstructed images of the contrast recovery phantom. The slices through the center of the spheres of the contrast recovery phantom are shown for ^99m^Tc and VXGP (A), ^90^Y 120–250 keV and MEGP (B), ^90^Y 120–250 keV and HEGP (C), ^90^Y 50–250 keV and MEGP (D), ^90^Y 50–250 keV and HEGP (E), ^166^Ho and MEGP (F), and ^166^Ho and HEGP (G). All images were linearly window-leveled from 0 to 4 times the average background intensity.

**Figure 5:**
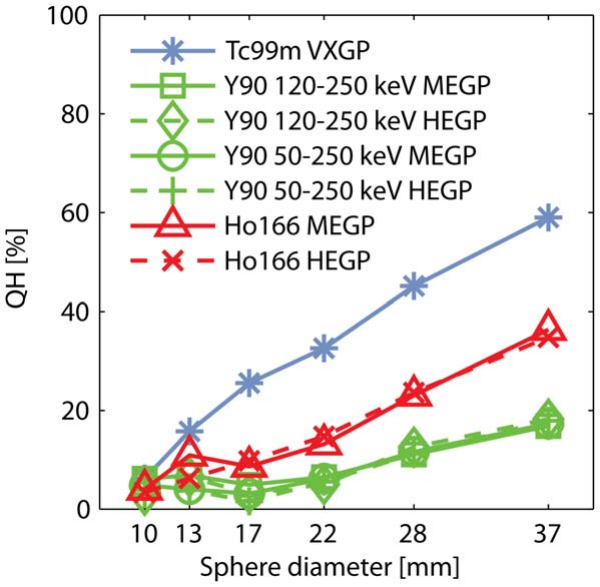
Contrast recovery as a function of sphere diameter. QH is the recovery of sphere-to-background contrast in the measurement, as compared to the true contrast in the phantom.

Figure 4 shows the slice through the centers of the spheres of all FBP reconstructed images. In Figure 5, contrast recovery coefficients are shown as a function of sphere diameter. The ^99m^Tc contrast recovery was 59% for the largest sphere. For ^90^Y and ^166^Ho, this was 17% and 35%, respectively. Neither collimator choice, nor energy window choice affected the ^90^Y contrast recovery much. Also, no difference was observed between the MEGP and HEGP ^166^Ho contrast recovery curves.

### Monte Carlo simulations

#### Primary, scatter, and penetration fraction

**Figure 6:**
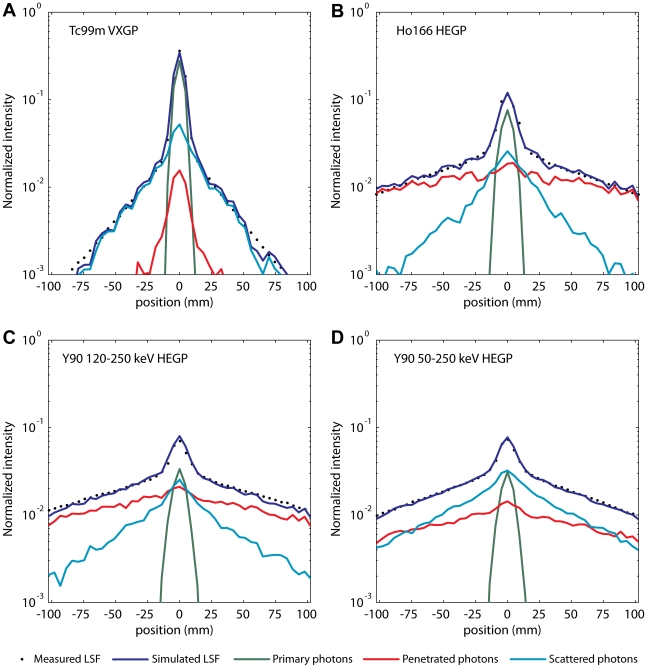
Measured and simulated LSF of the line-source centered in 20 cm PMMA. (A) LSF of the ^99m^Tc line-source and VXGP collimator, (B) ^166^Ho and HEGP, (C) ^90^Y 120–250 keV and HEGP, and (D) ^90^Y 50–250 keV and MEGP. Data is plotted on semi-logarithmic scale, showing good agreement between the measurements (data points) and simulations (blue solid line). The intensity is normalized to the total number of counts in the ROI. Contributions of primary, scattered, and penetrated photons are shown in green, light blue, and red, respectively.

The measured and simulated LSF of the line source centered in 20 cm PMMA are shown in Figure 6. The good agreement between both the center and the tails confirms the accuracy of the Monte Carlo simulations. The simulated LSF were subdivided in the three different photon classification groups. Table 6 lists the PF, SF and CPF of each isotope. For both ^90^Y and ^166^Ho, the HEGP collimator yielded a higher PF than the MEGP collimator, due to reduction of the number of penetrated photons. PF was similar for ^90^Y measured in the 120–250 keV window and in the 50–250 keV energy window, but the first was more contaminated by septal penetration, and the latter more by scatter. The ^166^Ho LSF was more contaminated by septal penetration than by scatter.

**Table 6:** Monte Carlo simulated primary, scatter and collimator penetration fractions for the experimental set-up with the line source centered in 20 cm PMMA.

	^99m^Tc	^90^Y 120–250 keV		^90^Y 50–250 keV		^166^Ho	
	VXGP	MEGP	HEGP	MEGP	HEGP	MEGP	HEGP
**PF [%]**	56	6	10	6	9	15	20
**SF [%]**	37	21	33	41	54	20	27
**CPF [%]**	7	73	58	52	37	65	54

## Discussion

The scintillation camera imaging characteristics of three isotopes used in RE were quantitatively evaluated with phantom experiments and Monte Carlo simulations. Although the superiority of ^99m^Tc was expected, its characteristics are valuable in evaluation of and comparison to the other two isotopes.

NEMA prescribes assessment of spatial resolution by means of point source measurements, but the more practical approach of the line source was chosen in this work. Nonetheless, good agreement between the FWHM presented here and in the literature was found for all isotopes. The measured FWHM of ^99m^Tc appeared to be slightly larger than that given in the specifications of the camera manufacturer (8.2 mm and 7.8 mm, respectively). This difference might be due to the measurement set-up with the source behind 1 cm PMMA. Indeed, measurement of the ^99m^Tc line source in air (data not shown) yielded a FWHM of 7.7 mm. The set-up was chosen, however, to be in line with ^90^Y and ^166^Ho measurements, which need the scatter material around the source to create all Bremsstrahlung photons. The spatial resolution of the ^90^Y LSF was in good agreement with results of previous studies, despite small differences in measurement settings. Shen et al reported a FWHM of 12.5 mm for a point source placed behind 1.8 cm of lucite, a source to detector (HEGP collimator) distance of 6.8 cm and an energy window of 55–285 keV [7], which is in good agreement with the FWHM of 11.4 mm found in this study for the line source behind 1 cm PMMA, a source to detector distance of 6 cm, and an energy window of 50–250 keV. Interestingly, the FWHM of the 120–250 keV window was slightly smaller than the 50–250 keV window, whereas the FWTM was substantially larger. This effect can be explained by the higher (collimator) scatter fraction in the 50–250 keV window, which broadened the FWHM, and the larger amount of collimator penetration in the 120–250 window, which caused a large FWTM. The spatial resolution of ^166^Ho was investigated by Bayouth and Macey, reporting a FWHM of approximately 16 mm of a line source measured behind 12 cm of scatter material [19], which is in good agreement with the FWHM of 15.6 mm of the ^166^Ho line source behind 10 cm PMMA (HEGP, collimator distance 11 cm) found in this study.

The ^90^Y planar system sensitivity was similar to earlier reports [7]. The sensitivity found for ^166^Ho was higher than the window-based, non-primary subtraction corrected sensitivity reported by Bayouth and Macey [19]. Good agreement was found if taking into account that about half of the detected photons of this study are non-primary, as was estimated from Monte Carlo simulations. The MEGP collimator showed a higher planar sensitivity than the HEGP collimator for both ^90^Y and ^166^Ho. However, Monte Carlo simulations elucidated higher primary fractions for the HEGP than for the MEGP collimator (Table 6). Monte Carlo simulations also revealed that ^90^Y and ^166^Ho non-primary photons lack spatial information, as is illustrated in Figure 6. Taking this into account, the useable sensitivity of the HEGP collimator, i.e. the sensitivity for primary photons, is similar to that of the MEGP collimator. With similar number of primary photons, and less background signal due to penetrated photons, the HEGP is preferred over the MEGP collimator for both ^90^Y and ^166^Ho.

The contrast recovery SPECT data was reconstructed using FBP, without inclusion of collimator modeling and scatter correction techniques. Compared to (model-based) iterative reconstruction algorithms, the FBP approach will result in suboptimal contrast recovery, but the first requires parameters that need to be optimized and have a large effect on image quality, such as the number of iterations. Reasonably accurate model-based quantitative image reconstruction methods have been proposed for both ^166^Ho and ^90^Y [5], [20], but these have distinct underlying algorithms, which makes straight-forward comparison of the isotope performance difficult. The FBP reconstructed contrast recovery coefficients reported in this study should not be interpreted as a measure of maximum isotope performance, but as a measure of isotope performance relative to each other. Since contrast recovery depends on resolution, scatter and penetration, it can be interpreted as a quantitative measure of overall image quality. This image quality of ^99m^Tc is higher than the ^166^Ho image quality, which in turn is higher than that of ^90^Y (Figure 5). No differences in ^90^Y contrast recovery were observed between energy windows and collimators. Although the HEGP collimator demonstrated a higher spatial resolutions and higher primary photon fraction than the MEGP collimator, differences were potentially too subtle to cause a substantial gain in contrast recovery in the FBP reconstructed images. Likewise, the ^166^Ho contrast recovery curves did not show differences between the MEGP and HEGP collimators. From Figure 4, however, it can be appreciated that the perceptual ^90^Y image quality is substantially better in the 50–250 keV energy window than the 120–250 keV energy window, due to reduction of the image noise. It can therefore be postulated that the use of a broad energy window and a HEGP collimator gave the optimal combination of sensitivity, spatial resolution and primary photon fraction for ^90^Y imaging, although differences with the MEGP collimator were small. The HEGP collimator also slightly outperformed the MEGP collimator in the ^166^Ho experiments.

The Monte Carlo simulations provided valuable information on the contribution and spatial distribution of different classes of photons, which could not be obtained by performing measurements alone. As is illustrated in Figure 6, there is a fundamental difference between the penetrated photons of ^99m^Tc and those of ^90^Y and ^166^Ho. Collimator penetration of ^99m^Tc photons is dominated by photons that slightly graze the septum edge. These photons maintain their spatial information. On the contrary, collimator penetration of ^90^Y and ^166^Ho is dominated by high-energy photons that, after penetration, scatter in the crystal or backscatter in the camera. These photons lack spatial information and degrade the image quality. Also, from Figure 6 it can be appreciated that photon scatter is the main source of degradation of ^90^Y image quality in the 50–250 keV energy window, and collimator penetration in the 120–250 keV energy window.

The results presented in this paper suggest that accurate scatter and collimator penetration correction techniques could greatly enhance the quantitative accuracy of ^90^Y and ^166^Ho scintillation camera imaging. Quantitative accuracies of ^99m^Tc SPECT with optimized correction techniques are typically within 5% for phantom studies and within 10% for clinical studies [31]–[34]. Reasonably accurate distribution estimates in phantoms have already been reported for ^166^Ho SPECT by de Wit *et al.* [20] and for ^90^Y by Minarik *et al.* [5]. The quantitative accuracy of ^99m^Tc SPECT might be approached if these correction schemes are further improved, which is important for accurate assessment of the distribution of the scout dose, determination of the lung-shunting fraction, and post-therapy liver dosimetry in radioembolization.

Besides scintillation camera imaging, other imaging modalities may also allow for quantitative assessment of the microsphere distribution in radioembolization. Recently, Positron Emission Tomography (PET) was proposed to image the ^90^Y therapy dose distribution [35]–[38] and phantom experiments have demonstrated the feasibility of quantitative imaging of the distribution of (non-radioactive) holmium with magnetic resonance imaging (MRI) [15], [39], [40]. Although both ^90^Y PET and holmium MRI have intrinsically promising imaging characteristics, such as a high spatial resolution, the clinical value of these modalities in microsphere radioembolization is yet unknown and subject of ongoing research.

In this study, the scintillation camera imaging characteristics of three isotopes used in radioembolization were quantitatively evaluated and compared. The use of a broad energy window and HEGP collimator gave optimal combination of sensitivity, spatial resolution, and primary photon fraction for ^90^Y Bremsstrahlung imaging, although differences with the MEGP collimator were small. For ^166^Ho imaging, the HEGP collimator slightly outperformed the MEGP. The image quality of both ^90^Y and ^166^Ho was affected more by partial volume effects, scatter, and collimator penetration than that of ^99m^Tc. The detailed breakdown of these imaging characteristics can help to direct the optimization of acquisition protocols and quantitative reconstruction algorithms for ^90^Y and ^166^Ho SPECT. The characterization of the three isotopes can guide future comparisons between or within clinical radioembolization studies with varying isotopes and imaging protocols.
